# Gas-Phase Synthesis of Dimethyl Carbonate from Methanol and Carbon Dioxide Over Co_1.5_pw_12_o_40_ Keggin-Type Heteropolyanion

**DOI:** 10.3390/ijms11041343

**Published:** 2010-03-31

**Authors:** Ahmed Aouissi, Zeid Abdullah Al-Othman, Amro Al-Amro

**Affiliations:** Department of Chemistry, King Saud University, P.O. Box 2455, Riyadh-11451, Saudi Arabia

**Keywords:** heteropolyanion, Keggin structure, methanol, dimethyl carbonate, direct synthesis, carbon dioxide

## Abstract

The reactivity of Co_1.5_PW_12_O_40_ in the direct synthesis of dimethyl carbonate (DMC) from CO_2_ and CH_3_OH was investigated. The synthesized catalyst has been characterized by means of FTIR, XRD, TG, and DTA and tested in gas phase under atmospheric pressure. The effects of the reaction temperature, time on stream, and methanol weight hourly space velocity (MWHSV) on the conversion and DMC selectivity were investigated. The highest conversion (7.6%) and highest DMC selectivity (86.5%) were obtained at the lowest temperature used (200 °C). Increasing the space velocity MWHSV increased the selectivity of DMC, but decreased the conversion. A gain of 18.4% of DMC selectivity was obtained when the MWHSV was increased from 0.65 h^−1^ to 3.2 h^−1^.

## 1. Introduction

Dimethyl carbonate (DMC) has drawn much attention in recent years as an environmentally friendly versatile intermediate. It has been used as a good solvent [1], an alkylation agent [2], and a substitute for highly toxic phosgene and dimethyl sulfate in many chemical processes [3,4]. In addition, it is expected to replace the gasoline oxygenate methyl *tert*. butyl ether (MTBE), because of its high oxygen content, low toxicity, and rapid biodegradability [1,5,6]. DMC has been produced by the reaction of methanol with phosgene in a concentrated sodium hydroxide solution [7]. However, owing to the high toxicity and the severe corrosivity of phosgene, this process has been abandoned gradually. Currently, DMC is produced mainly by oxidative carbonylation of methanol (non-phosgene route) [8]. The synthesis can be carried out in both liquid- and gas-phases. However, both routes use poisonous gas carbon monoxide and there is the possibility of an explosion. Recently, direct synthesis of DMC from CO_2_ and CH_3_OH has been reported as a most attractive route due to the low-cost of CO_2_ and the environmentally benign process [9–14]. However, DMC yield in this route is relatively low due to the fact that CO_2_ is thermodynamically stable and kinetically inert and due to the deactivation of catalysts induced by water formation in the reaction process [10,15]. Therefore, the development of efficient heterogeneous catalytic systems has attracted more attention. Bian *et al.* [16] studied the reaction over Cu–Ni/graphite nanocomposite catalyst in gaseous phase. They obtained 10.13% of CH_3_OH conversion and 89.04% of DMC selectivity at 105 °C. Wu *et al.* [17] studied the synthesis of DMC from gaseous methanol and CO_2_ over H_3_PO_4_ modified V_2_O_5_ catalyst with various molar ratios H_3_PO_4_/V_2_O_5_ (P/V). The best conversion (1.95%) and selectivity of DMC (92.12%) was obtained at 130 °C on the catalyst H_3_PO_4_/V_2_O_5_ (P/V = 0.20).

Here we report the direct synthesis of DMC from methanol and CO_2_ in gas phase over Co_1.5_PW_12_O_40_ as a Keggin-type heteropolyanion catalyst. The effect of the reaction temperature, MWHSV, and time on stream on DMC synthesis was investigated.

## 2. Results and Discussion

### 2.1. Characterization of Catalysts

**Figure 1.**
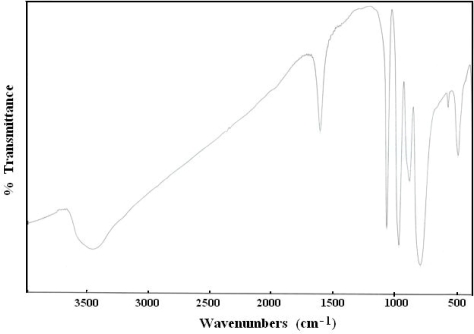
IR spectrum of Co_1.5_PW_12_O_40_ Keggin-type heteropolyanion.

**Figure 2.**
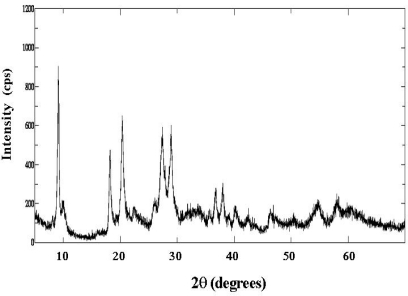
XRD pattern of Co_1.5_PW_12_O_40_ Keggin-type heteropolyanion.

The FT-IR spectrum of the Co_1.5_PW_12_O_40_ is shown in Figure 1. The IR spectrum has been assigned according to [18,19]. The main characteristic features of the Keggin structure are observed at 1080–1060 cm^−1^, 990–960 cm^−1^, 900–870 cm^−1^, and 810–760 cm^−1^, assigned to the stretching vibration ν_as_ (P-O_a_), ν_as_ (M-O_d_), ν_as_ (M-O_c_-M), and ν_as_ (M-O_c_-M), respectively (M = W or Mo). The result of X-ray powder diffraction of the Co_1.5_PW_12_O_40_ salt is shown in Figure 2. In each of the four ranges of 2θ, 7°–10°, 16°–23°, 25°–30°, and 31°–38°, the compound shows a characteristic for well defined Keggin structure of heteropolyanions [20–22]. So the presence of the primary Keggin structure in the synthesized phases was confirmed by FTIR and XRD.

#### Thermogravimetric (TG) and Dfferential Thermal Analysis (DTA)

**Figure 3.**
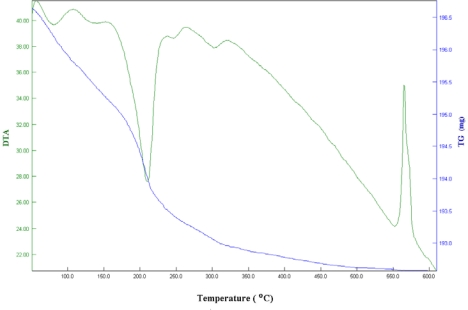
Differential thermal analysis (DTA) and thermo-gravimetric analysis (TGA) of Co_1.5_PW_12_O_40_ (heating rate: 5 °C/min).

Thermogravimetric analysis of Co_1.5_PW_12_O_40_ (Figure. 3) showed that the dehydration process starts at low temperature, about 50 °C, and finishes at 250 °C. The differential thermal analysis (DTA) indicates two endothermic peaks below 250 °C, and an exothermic peak at 575 °C. In agreement with the TG results, the endothermic peaks are assigned to the removal of physisorbed or crystallization water molecules (13–14 H_2_O), whereas the exothermic peak is due to the decomposition of Co_1.5_PW_12_O_40_ into the corresponding oxides (3/2CoO, 12 WO_4_ and 1/2P_2_O_5_). This result is in agreement with published results [23].

### 2.2. Catalytic Reaction

#### 2.2.1. Effect of Reaction Temperature

**Figure 4.**
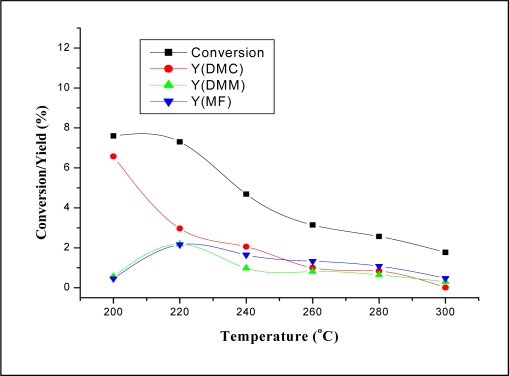
The dependence of the conversion and product yields on reaction temperature over Co_1.5_PW_12_O_40_. Reaction conditions: MWHSV = 3.25 h^−1^; molar ratio CH_3_OH/CO_2_ = 1.9.

**Figure 5.**
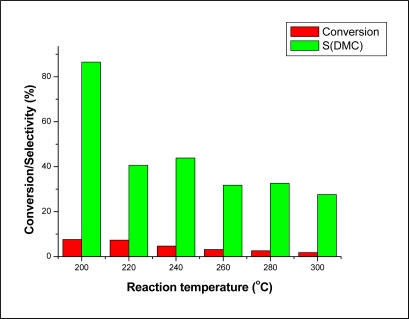
The dependence of the conversion and product selectivity on reaction temperature over Co_1.5_PW_12_O_40._ Reaction conditions: MWHSV = 3.25 h^−1^; molar ratio CH_3_OH/CO_2_ = 1.9.

The effect of the reaction temperature on the reaction performance was investigated at temperatures ranging from 200–300 °C. The results are illustrated in Figure 4 and 5. It can be seen from Figure 4 that CH_3_OH conversion and product yields decreased dramatically with increasing temperature. The decrease of both the conversion and product yields with increasing temperature might be due to the decreased CO_2_ adsorption on the catalyst at high temperatures [10]. Figure 5 shows that the selectivity of DMC decreased with increasing temperature. The decrease of DMC selectivity is probably due to the decomposition of DMC at higher temperatures [10,16, 24–26]. It can be seen from these results that the highest conversion of 7.6% and highest selectivity of DMC of 86.5% was obtained at the lowest temperature in this range of temperatures (200 °C).

#### 2.2.2. Effect of Time on Stream

**Figure 6.**
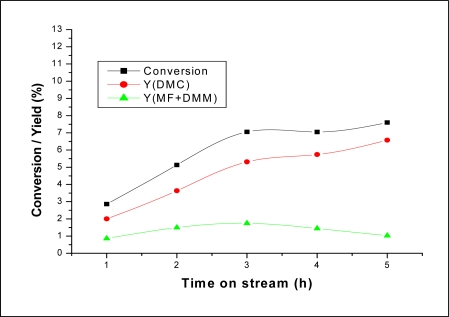
Time on stream effect on the conversion and product yields. Reaction conditions: T = 200 °C; MWHSV = 3.25 h^−1^; molar ratio CH_3_OH/CO_2_ = 1.9.

**Figure 7.**
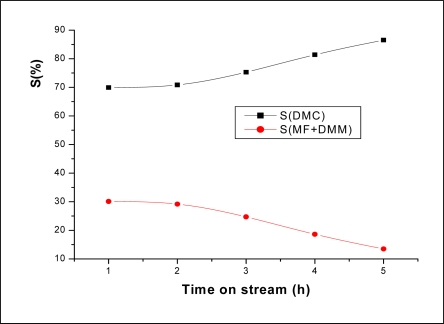
The product selectivities as a function of time on stream. Reaction conditions: T = 200 °C; MWHSV = 3.25 h^−1^; molar ratio CH_3_OH/CO_2_ = 1.9.

Figure 6 shows CH_3_OH conversion and products yield as a function of time on stream. It can be seen that both CH_3_OH conversion and DMC yield increased rapidly during the first three hours, then after increased slowly. DMM and MF were observed as minor products with a rate formation almost constant. As for the product selectivity, it can be seen from the figure 7 that DMC selectivity increased slightly with increasing time on stream to the detriment of DMM and MF.

#### 2.2.3. Effect of Space Velocity

**Figure 8.**
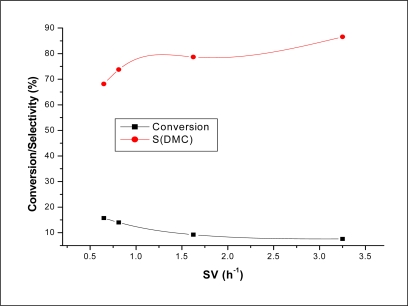
Conversion and DMC selectivity over Co_1.5_PW_12_O_40_ as a function of MWHSV. Reaction conditions: T = 200 °C; molar ratio CH_3_OH/CO_2_ = 1.9.

The effect of methanol weight hourly space velocity (MWHSV) on the conversion of methanol and the selectivity of DMC is shown in Figure 8. It can be seen that the conversion dropped sharply to about 6.4%. In fact, when the SV changed from 0.6 h^−1^ to 1.6 h^−1^, the conversion decreased from 15.7% to 9.3%. Further increase of MWHSV did not influence the conversion of methanol considerably. The conversion dropped only to about 1.7%. In fact, it has been found that when the MWHSV changed from 1.6 h^−1^ to 3.2 h^−1^, the conversion decreased from 9.3% to 7.6%. As for the selectivity of DMC, the result showed that increasing the MWHSV from 0.65 h^−1^ to 3.2 h^−1^ increased the selectivity from 68.1% to 86.5% gaining in this way 18.4% of DMC selectivity.

## 3. Experimental Section

### 3.1. Catalyst Preparation

The heteropolymolybdate salt namely Co_1.5_PW_12_O_40_ was prepared from 12-tungstophosphoric acid H_3_PW_12_O_40_ as precipitate by adding slowly the required amount of CoCO_3_. After protons neutralization by CO_3_^2−^, the cobalt salt, Co_1.5_PW_12_O_40_ was recovered from the solution by filtration

### 3.2. Physicochemical Techniques

The purity and the Keggin structure of the samples were characterized by means of IR and XRD. IR spectra were recorded with an infrared spectrometer GENESIS II-FTIR (400–4000 cm^−1^) as KBr pellets. The XRD powder patterns were recorded on a Rigaku diffractometer Ultima IV using CuKα radiation. Thermal analysis was carried out by means of differential thermal analysis (DTA) and thermogravimetric analysis (TGA) in air atmosphere with a 50 Shimadzu thermobalancesis

### 3.3. Catalytic Tests

The direct synthesis of DMC from methanol and CO_2_ was carried out at the temperature ranging from 200 °C to 300 °C in a flow-type fixed-bed stainless reactor loaded with 100 mg of catalyst under atmospheric pressure. To supply the reactant, a gas CO_2_ at a flow rate of 60 mL min^−1^ was passed through the methanol saturator thermostated at 40 °C. The molar ratio CO_2_/CH_3_OH was adjusted by the flux of CO_2_ controlled by mass flow controller and the temperature of saturator. Prior to the reaction, the catalyst was pretreated at 300 °C with CO_2_ for 2 h. The reaction products were analyzed with a gas phase chromatograph (Agilent 6890N) equipped with a flame ionization detector and a capillary column (HP-PLOT Q length 30m ID 0.53 mm). The condensed liquid was collected and analyzed. Catalytic activity was indicated by CH_3_OH conversion and product yields and selectivities. These parameters are calculated according to the following equations:

$${\text{Yield}}_{\left(\text{i}\right)}(\%)=\frac{{\text{N}}_{\text{i}}}{{\text{N}}_{\text{o}}}\times 100$$

$$\text{Conversion}\;(\%)=\frac{N_{r}}{N_{o}}\times 100=\sum {\text{Yield}}_{\left(\text{i}\right)}$$

$${\text{S}}_{\text{i}}(\%)=\frac{\text{Yield}(\text{i})}{\text{Conversion}}\times 100$$

N_o_: number of moles of methanol introduced (mol/h)
Ni: number of moles of the product i formed (mol/h)
N_r_: number of moles of reacted methanol (mol/h)

## 4. Conclusion

Synthesis of DMC from CH_3_OH and CO_2_ has been studied in gas phase system under atmospheric pressure using Co_1.5_PW_12_O_40_ as a catalyst. It was found that both CH_3_OH conversion and DMC yield decreased with increasing temperature, owing to the decreased CO_2_ adsorption on the catalyst at high temperatures. As for the decrease of DMC selectivity, this is probably due to the decomposition of DMC at higher temperatures.

Lower temperature leads to high selectivity of DMC to the detriment of that of DMM and MF. The optimal reaction conditions for the synthesis of DMC is lower temperature and high space velocity.
